# SLC45A3 Serves as a Potential Therapeutic Biomarker to Attenuate White Matter Injury After Intracerebral Hemorrhage

**DOI:** 10.1007/s12975-023-01145-5

**Published:** 2023-03-13

**Authors:** Yi Zhang, Hanhai Zeng, Feiyang Lou, Xiaoxiao Tan, Xiaotong Zhang, Gao Chen

**Affiliations:** 1 Department of Neurosurgery, The Second Affiliated Hospital of Zhejiang University School of Medicine, Zhejiang University, Hangzhou, 310016 China; 2 Key Laboratory of Precise Treatment and Clinical Translational Research of Neurological Diseases, Hangzhou, 310016 China; 3 The Interdisciplinary Institute of Neuroscience and Technology, Zhejiang University, Hangzhou, 310020 China; 4 College of Electrical Engineering, Zhejiang University, Hangzhou, 310027 China; 5 MOE Frontier Science Center for Brain Science and Brain-machine Integration, Zhejiang University, Hangzhou, 310058 China

**Keywords:** Intracerebral hemorrhage, Weighted gene co-expression networks analysis, Differentially expressed genes, Single-cell RNA sequencing, Diffusion tensor imaging

## Abstract

Intracerebral hemorrhage (ICH) is a severe cerebrovascular disease, which impairs patients’ white matter even after timely clinical interventions. Indicated by studies in the past decade, ICH-induced white matter injury (WMI) is closely related to neurological deficits; however, its underlying mechanism and pertinent treatment are yet insufficient. We gathered two datasets (GSE24265 and GSE125512), and by taking an intersection among interesting genes identified by weighted gene co-expression networks analysis, we determined target genes after differentially expressing genes in two datasets. Additional single-cell RNA-seq analysis (GSE167593) helped locate the gene in cell types. Furthermore, we established ICH mice models induced by autologous blood or collagenase. Basic medical experiments and diffusion tensor imaging were applied to verify the function of target genes in WMI after ICH. Through intersection and enrichment analysis, gene SLC45A3 was identified as the target one, which plays a key role in the regulation of oligodendrocyte differentiation involving in fatty acid metabolic process, etc. after ICH, and single-cell RNA-seq analysis also shows that it mainly locates in oligodendrocytes. Further experiments verified overexpression of SLC45A3 ameliorated brain injury after ICH. Therefore, SLC45A3 might serve as a candidate therapeutic biomarker for ICH-induced WMI, and overexpression of it may be a potential approach for injury attenuation.

**Graphical Abstract:**

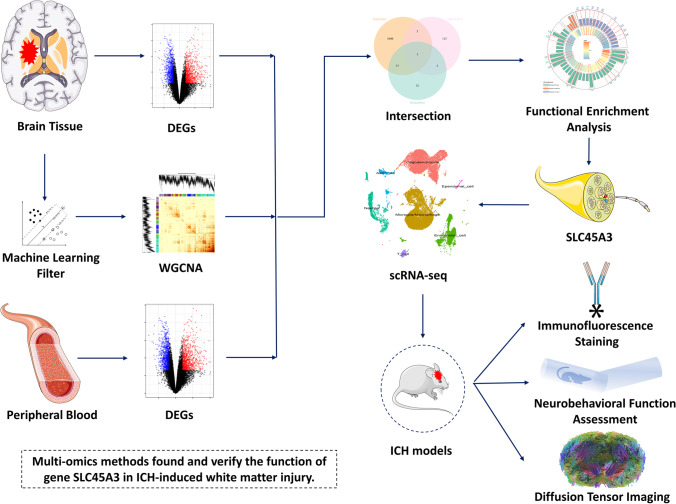

**Supplementary Information:**

The online version contains supplementary material available at 10.1007/s12975-023-01145-5.

## Introduction

Intracerebral hemorrhage (ICH) is an emergent and critical cerebrovascular disease, with high mortality and disability rates [1]. For most patients, various degrees of neurological dysfunctions will persist in their later lives even after aggressive treatments. The primary injury is caused by the massive hematoma itself, whereas the secondary damage results from multiple mechanisms (such as neuroinflammation, cytotoxicity of erythrocyte lysates, neurotoxicity of thrombin, and oxidative stress), which are strongly associated with the regulation of gene expression levels [2, 3]. Some researchers, therefore, investigated the gene expression profiles after ICH, discovering several interesting candidate genes (e.g., C3AR1, ARNTL2, ETS1, and LCP1) involved in the abovementioned mechanisms [4–7], with the rapid advance of high throughput sequencing and bioinformatics. For example, it was reported that the genes regulating peripheral immune cells infiltration may make a difference in brain injury induced by ICH [6].

More recently, instead of focusing on these pathophysiological processes (e.g., death of neurons, neuroinflammation, and gray matter damage), white matter injury (WMI) after ICH has attracted more attentions. It plays a significant role in neurological rehabilitation of ICH, since damage to these neural fibers negatively influences patients’ motor, sensory, and cognitive functions directly, thus being a potential treatment target as indicated by previous studies [8–11]. It was suggested that the process of demyelination, axonal damage, and mature oligodendrocyte loss may all be involved in ICH-induced WMI [12]. The information about the underlying mechanism and pertinent treatment, however, is still insufficient yet. To investigate the WMI, in addition to traditional basic medical experiments (e.g., immunohistochemical staining, transmission electron microscopy, and the neurofilament light chain), currently, magnetic resonance imaging (MRI) could provide a promising method to detect white matter structure in vivo by the technique of diffusion tensor imaging (DTI), as metrics obtained through DTI such as fractional anisotropy (FA), apparent diffusion coefficient (ADC), and mean diffusivity (MD) are effective to detect the integrity of white matter[13, 14]. In most DTI research, the FA value has been reported to be a reliable and stable indicator to reflect WMI [15, 16].

In this study, we used multiple bioinformatics algorithms including weighted gene co-expression networks analysis (WGCNA) [17], Gene Ontology (GO) terms, and Kyoto Encyclopedia of Genes and Genomes (KEGG) pathway enrichment analysis, as well as single-cell RNA sequencing (scRNA-seq) to find interesting genes related to WMI after ICH. Furthermore, after established ICH mice models induced by autologous blood or collagenase, we carried out experiments about immunohistochemical staining, western blotting, neurobehavioral function assessment, and DTI scanning in vivo to verify the function of discovered gene in WMI after ICH. This study aims at portraying the WMI condition after intervention during period of ICH, thus extending our understanding on ICH and hopefully would inspire innovative therapeutic targets for ICH-induced WMI.

## Materials and Methods

### Datasets Information and Pre-processing

The data were obtained from the Gene Expression Omnibus (GEO) database. A microarray dataset, GSE24265, includes brain samples (e.g., perihematomal areas, contralateral grey matter, and contralateral white matter) from deceased patients who suffered from ICH [4]. To remove the batch effects, the “sva” package in R language (version 4.1.0) was applied [18]. Another dataset, GSE125512, contains the RNA-sequencing results of peripheral blood sample of patients with ICH and was collected within 24 h of symptom onset and after 72 h following the first, respectively [5]. Single-cell RNA-seq dataset, GSE167593, including expression profiling from 8 control mice and 8 hemorrhagic stroke mice, was obtained from striatum tissue [19].

### Construction of Weighted Gene Co-expression Networks

Constructing weighted gene co-expression networks was accomplished by the “WGCNA” package (version 1.71) in R language [17]. A machine-learning algorithm, called support vector machine (SVM), was used by the “scikit-learn” package (version 1.1.1) in Python to filter out those genes with minimum within-group variance [20], since they can be regarded as the background noise according to WGCNA algorithm. In SVM, cross-validation was achieved by the leave-one-out method. Accuracy and F1 score were adopted as the performance measures, and the two statistical indicators were defined as Eqs. (1) and (2) in our leave-one-out method, respectively:

1 $$Accuracy= \frac{1}{m} \sum_{i=1}^{m}I \left(f\left({x}_{i}\right)= {y}_{i}\right)$$

2 $$F1 score= \frac{1}{m} \sum_{i=1}^{m}(\frac{2 \times {TP}_{i}}{2 \times {TP}_{i}+{FN}_{i}+{FP}_{i}})$$

in which *m* represents the number of loops in leave-one-out method, 𝕀() denotes the indicator function which returns 1/0 if the input is TURE/FALSE, $f\left({x}_{i}\right)$ denotes the predicted labels given by SVM whereas ${y}_{i}$ denotes the true label; TP, FN, and FP mean the number of true positive, false negative, and false positive in confusion matrix, respectively.

After filtering, the function “pickSoftThreshold” picked a thresholding power β, according to which a topological overlap matrix (TOM) was constructed with the parameters as follows: minModuleSize = 80, mergeCutHeight = 0.20. In TOM, these genes were separated into diverse modules, where module eigengenes (MEs) were defined as the first principal components of each gene module considered as a representation of the gene expression profiles in this module. Then, the correlation between modules and clinical traits was calculated. Those genes in the most correlated modules were extracted to compute their module membership (MM) and gene significance (GS). Here, MM means the correlation between the expression profile of one gene and the ME, while GS was defined as minus log *p*-value of each gene. In the last, those with MM > 0.9 and GS > 0.85 were regarded as interesting genes.

### Dataset Analysis

For GSE24265, identification of the differentially expressed genes (DEGs) was performed by “limma” package in R language [21] whereas DEGs had already been identified in the downloaded series matrix files of GSE125512. The threshold of fold change was set as > 2 or <  − 2 while the one of false discovery rate (FDR) was adopted as 0.05 with the Benjamini–Hochberg method after *t* test. The intersection of DEGs in the two datasets was further carried out with Gene Ontology (GO) terms and Kyoto Encyclopedia of Genes and Genomes (KEGG) pathway enrichment analysis through “clusterProfiler” package in R language [22]. Respectively, the enrichment results of three processes (i.e., biological process (BP), cellular component (CC), and molecular function (MF)) were gathered and visualized by “GOplot” package [23]. Moreover, another intersection among the interesting genes found by WGCNA, the DEGs in GSE24265 and GSE125512 were performed to find the target gene, and its related pathways in GO analysis results were visualized by “circlize” package [24]. For scRNA-seq analysis, gene counting was accomplished by “Cell Ranger” (version 7.0.1) software in Linux, while batch effect removing and cluster dividing were achieved by “Seurat” package (version 4.2.1)[25], and cell type annotation by “SingleR” package [26].

### Animals

C57BL/6 mice (6–8 weeks, 20–25 g) (SLAC Laboratory Animal Company, Shanghai, China) used in the present study were all male for the purpose of diminishing the bias from estrogen. Animals were housed in a comfortable environment with temperature and humidity regulated carefully, a 12-h light–dark cycle established suitably, food and water supplied ad libitum.

Referring to former research, an ICH model construction procedure was performed [27, 28]. Particularly, after anesthetization by pentobarbital sodium (40 mg/kg, 1%) through intraperitoneal injection, mice were injected with 25 µl whole blood (from the tail artery) or 0.05 U type VII collagenase (Sigma-Aldrich, St. Louis, MO) blended in 0.5 µl saline according to their groups (autologous whole blood injection groups or collagenase injection groups). Stereotactic instrument (RWD Life Sciences Ltd. Shenzhen, China. Code: 68,535) was applied to ensure the position of injection (right basal ganglion) following the protocol (2.5-mm lateral to the bregma, 3-mm deep at a 5° angle). Throughout the whole process, body temperature was maintained at 37 ± 0.5 °C by monitoring rectal temperature. The same operation was carried out in sham mice, which included needle insertion but no blood or collagenase injection.

### AAV Construction

The serotype of AVV was AVV9. To construct the SLC45A3-AAV, the cDNA encoding human SLC45A3 gene (primers: 5′-TTTGGCAAAGAATTGGATCCCGCCACCATGATCCAGAGGCTGTGGGC-3′ and 5′-AGTCCATGGTGGCGACCGGCACTGAGTATTTGGCCAAGTCGTTC-3′) was amplified. It was thereafter inserted between the BamHI and AgeI sites of vector GV634 (Genechem Company, Shanghai, China).

### Drug Administration

At 3 weeks before ICH induction, scrambled NC-AAV (1.24e + 12 vg/ml, 3 μl) or SLC45A3-AAV (1.21e + 12 vg/ml, 3 μl) (Genechem Company, Shanghai, China) was infused into the right lateral ventricle, with mice kept anesthetized by pentobarbital sodium as described above. Then, a small burr hole (1 mm posterior to the bregma and 1.0 mm right lateral to the midline) was drilled carefully, through which the drugs were administrated slowly (3.2 mm in depth, 0.5 μl/min). After 5 min remained in place, the needle was gently withdrawn over another period of 5 min. Finally, bone wax and sutures were utilized to close the burr hole and stitch the incision, respectively. All of the surgical procedures were performed under sterile conditions.

### Assessment of Neurobehavioral Function

The assessments of neurobehavioral function were executed by two investigators blinded to examining conditions. In total, 3 distinct tests (i.e., forelimb placing test, corner turn test, and cylinder test) were conducted to estimate their neurobehavioral functions at days 1, 3, 5, 7, and 14 after the onset of ICH [29]. Precisely, in forelimb test, for each mouse, its torso was constrained whereas its forelimb was allowed to hang freely. Each test was repeated 10 times, and for all trials, the percentage of whether the mouse put its correct forelimb on the worktop responding to vibrissae stimulation was documented. In corner turn test, two plastic walls made up a 30° corner where each mouse could turn either right or left freely to depart this corner. As above, repetition time was 10 to record the proportion of right turn times. For cylinder test, in a transparent cylinder (height: 25 cm and inner diameter: 8 cm), the mouse was permitted to rear freely for 20 times. In each trial, the forelimb used to help rear against the wall was documented (*R* denotes right forelimb, *L* denotes left forelimb, and *B* denotes both forelimbs). The calculation of scores was as follows: score = ((*R* − *L*)/(*R* + *L* + *B*)) × 100%, and herein, a higher score indicated worse neuronal function.

### Western Blotting

Western blotting was conducted using samples from right basal ganglia area as previously described [30]. Sample proteins were firstly lysed by RIPA lysis buffer. After the protein (40 μg) was separated by 10% SDS-PAGE, it was transferred to PVDF membranes. Skim milk (5%) was applied to block the PVDF membranes at room temperature for 1 h and thereafter incubated overnight at the temperature of 4 °C with the following primary antibodies: SLC45A3 antibody (1:1000, Abcam, ab137065) and β-actin antibody (1:5000, Abcam, ab8226). The PVDF membranes were incubated with horseradish peroxidase conjugated secondary antibodies for 1 h at room temperature. The ECL Plus chemiluminescence reagent kit (Amersham Bioscience, Arlington Heights, IL, USA) was used to visualize the blots while ImageJ was utilized for protein quantification.

### Immunofluorescence Staining

Immunofluorescence staining was performed the same way as previously implemented [30]. Firstly, after anesthetizing, the mouse was perfused through ice-cold PBS (0.1 mol/L, 20 mL) via its cardiac apex, followed by another perfusion with 4% paraformaldehyde (PFA). Secondly, the whole brain was extracted and immersed by 4% PFA overnight and was thereafter immersed in 30% sucrose at 4 °C for 72 h. Thirdly, for subsequent experiments, the brains were cut into coronal slices (8 μm) and fixed onto glass slides. After blocking by 5% BSA and 0.3% Triton X-100, these coronal slices were then incubated with primary antibodies overnight at 4 °C. The information of antibodies was as follows: SLC45A3 antibody (1:100, Abcam, ab137065) and MBP antibody (1:500, Cell Signaling Technology, CST#83683S). Fourthly, the sections were incubated by secondary antibodies at 37 °C for 2 h. The secondary antibodies were Alexa Fluro 488-conjugated donkey anti-mouse IgG (1:500, Invitrogen, A21202) and Alexa Fluro 555-conjugated donkey anti-rabbit IgG (1:500, Invitrogen, A32794). Finally, for each mouse, 5 sample slices were examined, and 3 fields of view were chosen per slice to calculate the mean number of target cells by a fluorescence microscope (Leica, Mannheim, Germany).

### Diffusion Tensor Imaging and Data Processing

All magnetic resonance imaging scans were performed on a 7.0 T pre-clinical animal scanner (BioSpec 70/16 USR, Bruker BioSpin MRI, Ettlingen, Germany) with a Bruker quadrature birdcage volume coil (T20061V3). Through the scanning, each mouse was anesthetized by 1–3% isoflurane mixed with compressed air (1.5L/min). The body temperature was maintained at 37 °C, and respiratory rate was monitored. Mice were scanned with Rapid Acquisition with Relaxation Enhancement (RARE) for T2-weighted imaging: TR/TE = 2500 ms/8.75 ms, matrix size = 256 × 256, FOV = 18 × 18 mm^2^, slice thickness = 0.5 mm, excitation flip angle = 90°, refocusing flip angle = 180°, 2 averages, scan time 2′40″ and Fast Low-angle Shot (FLASH) for T1-weighted imaging: TR/TE = 264.6 ms/4 ms, matrix size = 256 × 256, FOV = 18 × 18 mm^2^, slice thickness = 0.5 mm, excitation flip angle = 30°, 2 averages, scan time 1′42″. Diffusion tensor imaging (DTI) was performed by an echo-planar imaging (EPI) sequence: TR / TE = 3000 ms / 23.54 ms, matrix size = 96 × 96, FOV = 18 × 18 mm^2^, slice thickness = 0.5 mm, flip angle = 90°, scan time 13′00″. *B* values were 1000 and 2000s/mm^2^ in 30 directions while five additional images were also acquired at *b* = 0 s/mm^2^.

Analysis of the diffusion tensor imaging data was accomplished by DSI Studio software [31]. Firstly, bruker “2dseq” files were reconstructed into DTI data. Secondly, diffusion tensor eigenvalues (λ1, λ2, and λ3) were computed according to DTI data, *b* values, and directions. Secondly, fractional anisotropy (FA), an index indicating the integrity of white matter, was calculated as stated by Eq. (3), in which $\overline{\lambda }$ equals the mean value of the eigenvalues (that is, $\overline{\lambda }$ = (*λ*1 + *λ*2 + *λ*3)/3):

3 $$FA= \sqrt{\frac{3}{2}} \frac{\sqrt{{\left({\lambda }_{1}-\overline{\lambda }\right)}^{2}+{\left({\lambda }_{2}-\overline{\lambda }\right)}^{2}{\left({\lambda }_{3}-\overline{\lambda }\right)}^{2} }}{\sqrt{{\lambda }_{1}^{2}+ {\lambda }_{2}^{2}+ {\lambda }_{3}^{2}}}$$

Thirdly, regions of interest (ROIs) were delineated with the same voxel sizes (3 × 3 × 3), in order to lessen the influence from different volume sizes. Referring to standard template (“CIVM_mouse”) and colored FA map, ROIs were carefully outlined in the ipsilateral and contralateral side. In each side, 15 ROIs were drawn in the corpus callosum (CC) whereas 10 ROIs for internal capsule (IC). The definition of this kind resulted in 9 voxels in plane and through 3 slices per ROI, and the mean FA ($\overline{FA }$) in each ROI was figured out as claimed by Eq. (4), in which *n* denotes the number of voxels in plain and *m* means the number of slices. Then in each ROI, $\overline{FA }$ in right side divided by the one in left gave the ratio of FA, according to their corresponding positions:

4 $$\overline{FA }=\frac{1}{m}\frac{1}{n} \sum_{i=1}^{m}\sum_{j=1}^{n}{FA}_{ij}$$

Attempt for fiber tracking was made in self-defined ROI (hematoma been delineated then dilated by 5 voxels) by DSI Studio software, with tracking parameters set as angular threshold = 30°, step size = 0.05 mm, tracking seeds = 1,000,000.

### Statistical Analysis

For basic medical experiments (neurobehavioral function test and immunofluorescence staining), continuous data are exhibited with mean ± standard deviation (SD) or median (interquartile range) according to their normality and homogeneity of variance. For those with a normal distribution, Student’s *t* test (2 groups) and one-way analysis of variance (ANOVA) (≥ 3 groups) were performed to analyze significant differences. For those failed to distribute normally, statistical analysis was carried out through the Mann–Whitney *U* test (2 groups) or Kruskal–Wallis test (≥ 3 groups). Post hoc comparisons were performed through Fisher’s least significant difference (LSD) test. For neurobehavioral function assessment, statistical analysis among groups was conducted at the same time point. A *p*-value < 0.05 suggested statistical significance. GraphPad Prism (Version 9.4.1) and SPSS software (Version 23.0) were utilized for this part of statistical analysis with blind method.

For MRI data, statistical analysis was accomplished by R language. Within 3 groups (Sham group, ICH + NC-AAV group, and ICH + SLC45A3-AAV group), each consisted of the figures of 6 subgroups: FA values in right CC, FA values in left CC, FA ratios in CC, FA values in right IC, FA values in left IC, and FA ratios, which generated 18 subgroups in total. Instead of simply using mean ± standard deviation (SD) or median (interquartile range) from raw data to represent results of subgroups, a remarkably more accurate method, the bootstrap method, was applied to produce 95% confidence intervals (CI) [32]. This process was accomplished by “boot” package [33] in R with its resampling times set as 10,000. Comparisons among subgroups with the same data type (e.g., FA values in right CC among Sham group, ICH + NC-AAV group, and ICH + SLC45A3-AAV group) were achieved by a superior statistical method, Fisher-Pitman Permutation Test [34]. It is an exact statistic hypothesis test (or called re-randomization test) which uses the proof on the opposite where the distribution of statistical indicators under given null hypothesis is acquired by computing all possible numbers of these statistic tests through attainable rearrangements of real observed data [35]. This procedure was performed by “coin” package [36] in R language with its resample times adjusted as 10,000 via Monte Carlo resampling. Comparisons between two subgroups were carried out if there was statistically significant difference among three subgroups (e.g., FA values in right CC among Sham group, ICH + NC-AAV group, and ICH + SLC45A3-AAV group). Statistically significant difference was considered at *p-value* < 0.05. The results of statistical analysis were visualized by “circlize” package [24] in R language.

### Ethics Statement

Animal experimentation was approved by the Institutional Animal Ethics Committee of Zhejiang University and performed in accordance with the National Research Council Guide for Care and Use of Laboratory Animals.

## Results

### WGCNA Analysis Determined the Genes Relevant to ICH

**Fig. 1:**
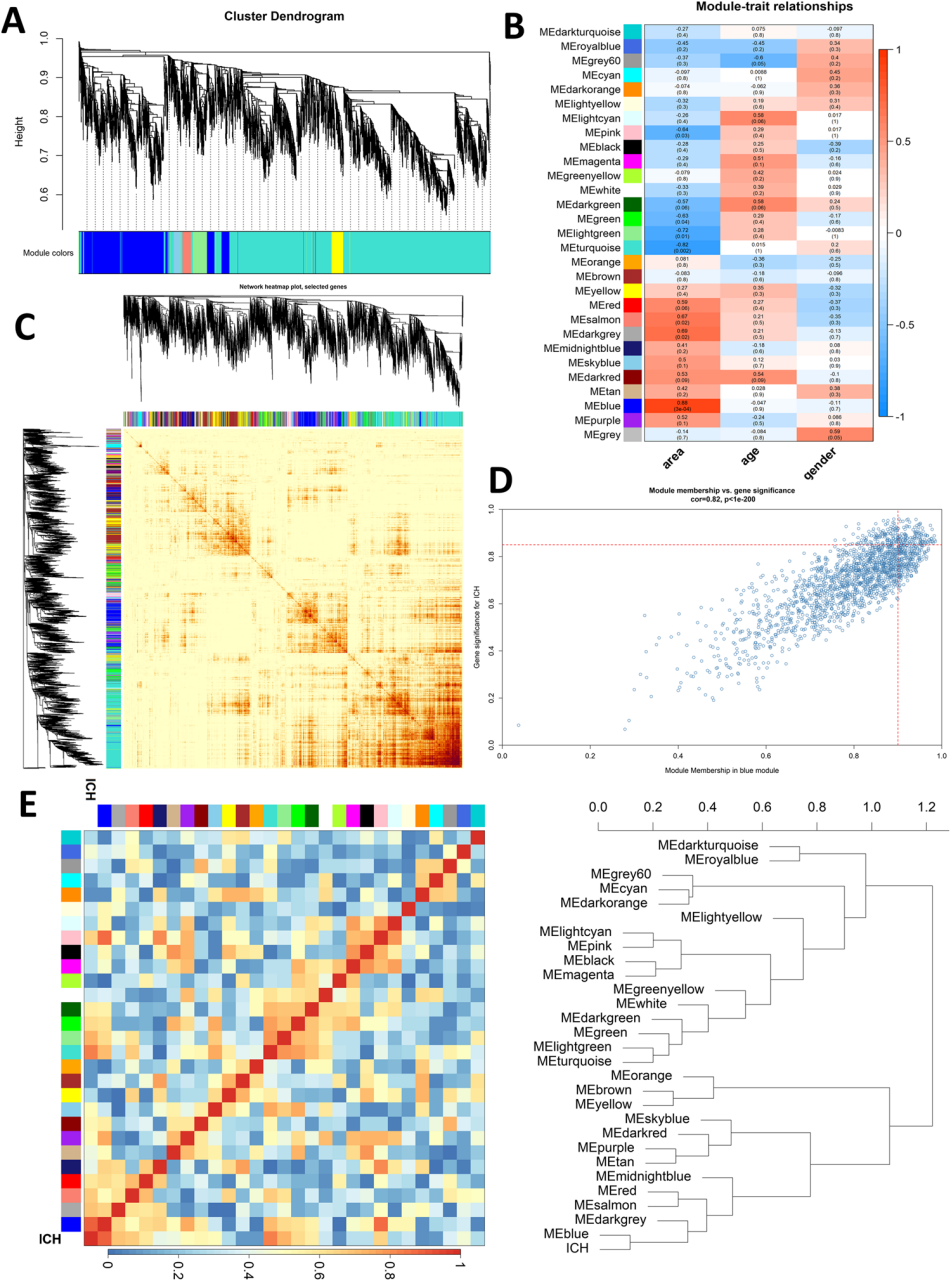
Construction of co-expression networks by WGCNA. **A** Results of cluster dendrogram creation. The branches above stand for genes and different colors represent distinct co-expression modules. **B** Module-trait correlation matrix among gene modules. **C** TOM showed via heatmap (5000 genes). **D** MM vs. GS in blue module. The red dashed lines illustrate the thresholds of MM (> 0.9) and GS (> 0.85) set for interesting genes. **E** Dendrogram of eigengenes and adjacency heatmap additionally demonstrates the correlation between modules and ICH

Graph theory demonstrates the ubiquity of small-world network, in which several hub nodes take the responsibility of communications between modules as well as the correlation patterns of gene expression [17]. In our study, 30% genes with minimum within-group variance were filtered out according to the performance measures of machine learning algorithm (Supplementary Fig. 1A). The soft thresholding power β was set to 12 with the topological *R*^2^ equaling 0.86 to construct a scale-free network (Supplementary Fig. 1B), which resulted in a network containing twenty-nine modules illustrated by various colors (Fig. 1A). To assess which module is most tightly associated with ICH, a module-trait correlation matrix was built (Fig. 1B, “area” represents ipsilateral perihematomal areas or contralateral areas)). The blue module was found to be the most relevant one. Furthermore, gene dendrogram with adjacency matrix for overall genes (5000 genes randomly sampled), GS vs. MM in blue module, and network heatmap were plotted to show the relationships in blue module (Fig. 1C–E). Finally, those with MM > 0.9 and GS > 0.85 in blue module were regarded as interesting genes (94 genes were selected in total, Fig. 1D).

### Differentially Expressed Genes and Enrichment Analysis Indicated Gene SLC45A3

**Fig. 2:**
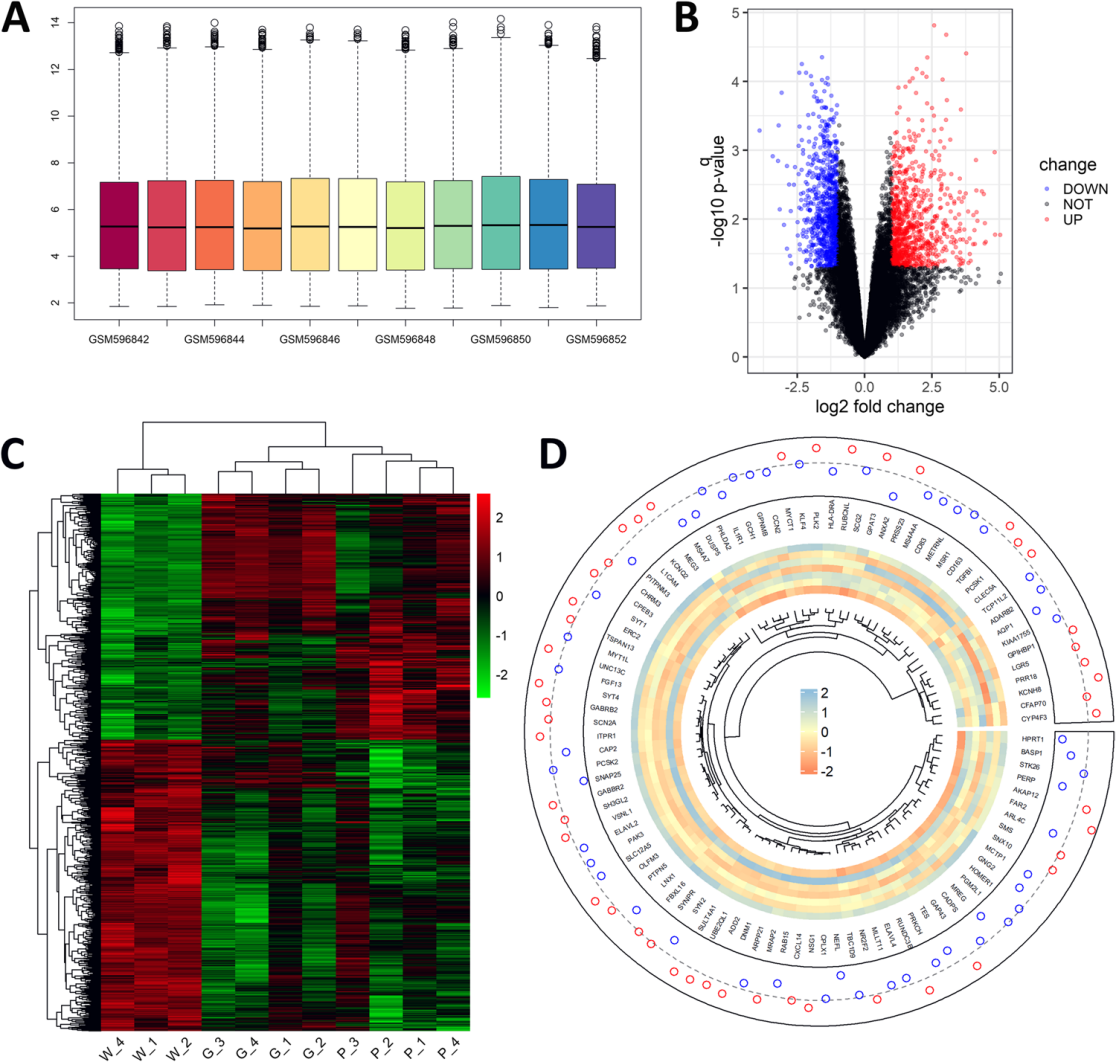
Differentially expressed genes (DEGs) in GSE24265. **A** Boxplot shows the average gene expression levels after normalization. **B** Volcano plot indicates the distribution of the DEGs (red: upregulated genes; blue: downregulated genes). **C** Heatmap of overall gene expression levels (red: upregulated genes; green: downregulated genes). **D** Map of the top 100 most differentially expressed genes ranked by fold change between perihematomal tissues and contralateral white matter. In points plot, the dashed grey line represents 0 and points stand for average expression levels (blue: below 0; red: above 0). In heatmap, red represents upregulated genes, and blue represents downregulated genes

Figure 2A illustrates the gene expression levels of different samples in dataset GSE24265 after normalization. With the threshold set as q-values (*p*-values after false discovery rate correction) < 0.05 and | fold change |> 2, a total of 1105 DEGs were identified in the volcano plot (Fig. 2A). Figure 2C exhibits the overall gene expression levels in three different tissues (ipsilateral perihematomal tissues (P_1-4), contralateral white matter (W_1-3), and contralateral gray matter (G_1-4)). Figure 2D exemplifies the top 100 DEGs between perihematomal tissues and contralateral white matter according to their fold change. The same procedure was applied in dataset DSE125512, helping to find 125 DEGs. By taking an intersection between the DEGs of GSE24265 and GSE125512, only 8 candidate genes were screened out (OLIG2, MGLL, CMTM5, COL9A2, PCOLCE2, SDC2, SLC45A3, RHOBTB1)

**Fig. 3:**
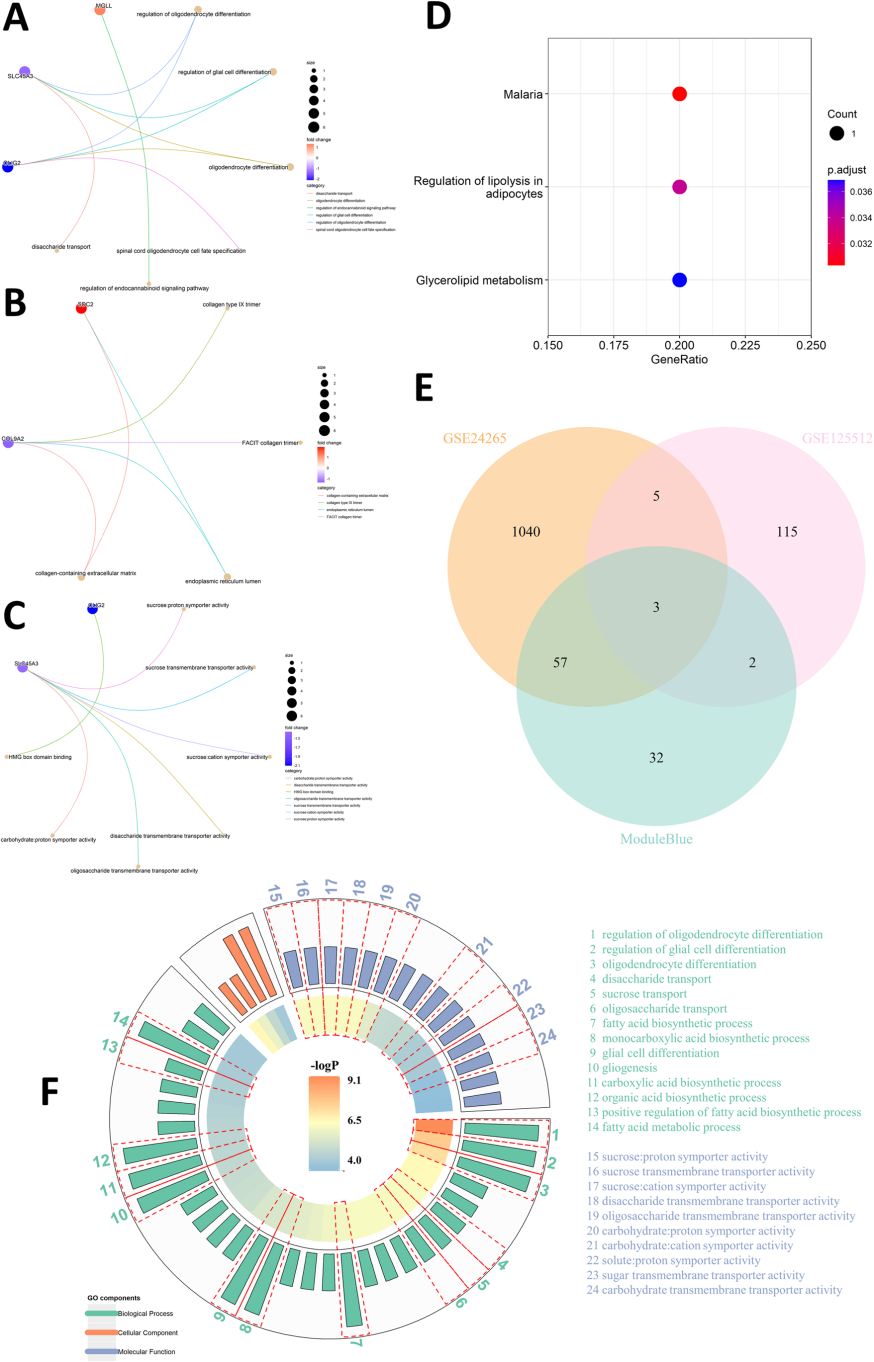
Enrichment analysis of GO and KEGG in candidate genes. **A**–**C** Enrichment components in GO biological process, cellular component, and molecular function, respectively. The golden nodes denote GO terms while other colorful ones represent related genes. **D** Enriched KEGG pathways. **E** Venn plot displays the intersection among interesting genes found in WGCNA, DEGs in GSE24265, and GSE125512. **F** A summary of GO enrichment analysis. The different colors in the outer annulus represent three components of GO and the inner colorbar indicates − logP values. Those related to gene SLC45A3 are circled by red dashed lines and listed in the right

Analysis of functional enrichment was carried out in candidate genes, including GO and KEGG analysis. Figure 3A–C show the enrichment outcomes of biological process, cellular component, and molecular function, respectively. Figure 3D displays the enrichment results of KEGG inquiry, suggesting these candidate genes are mainly abundant in pathways of glycerolipid metabolism and regulation of lipolysis in adipocytes. Another intersection between the eight candidate genes and interesting genes found in WGCNA was conducted (Fig. 3E), obtaining 3 genes ultimately: CMTM5, COL9A2, and SLC45A3.

In the final 3 genes, given that SLC45A3 was notably found in both BP and CC parts in GO analysis, it was eventually selected as our target gene. As is illustrated in Fig. 3F, a summary of functional enrichment of gene SLC45A3 in GO analysis indicates that gene SLC45A3 may have functions of regulation of oligodendrocyte differentiation, involving in fatty acid metabolic process, etc. after ICH.

### The scRNA-seq Analysis Revealed SLC45A3 Mainly Located in Oligodendrocytes

Cluster analysis through uniform manifold approximation and projection (UMAP) disclosed that the cells from both control and ICH groups could be divided into 7 clusters: “Oligodendrocyte,” “Microglia/Macrophage,” “Endothelial_cell,” “Neuron,” “Astrocyte,” “T_cell,” “Ependymal_cell,” and “Neuron” (Supplementary Fig. 2A). Although all cell types more or less expressed SLC45A3, it was evident that SLC45A3 mainly expressed in oligodendrocytes, and the expression decreased in ICH groups (Supplementary Fig. 2B).

### SLC45A3 Expression in the Brain Decreased After ICH in Mice

**Fig. 4:**
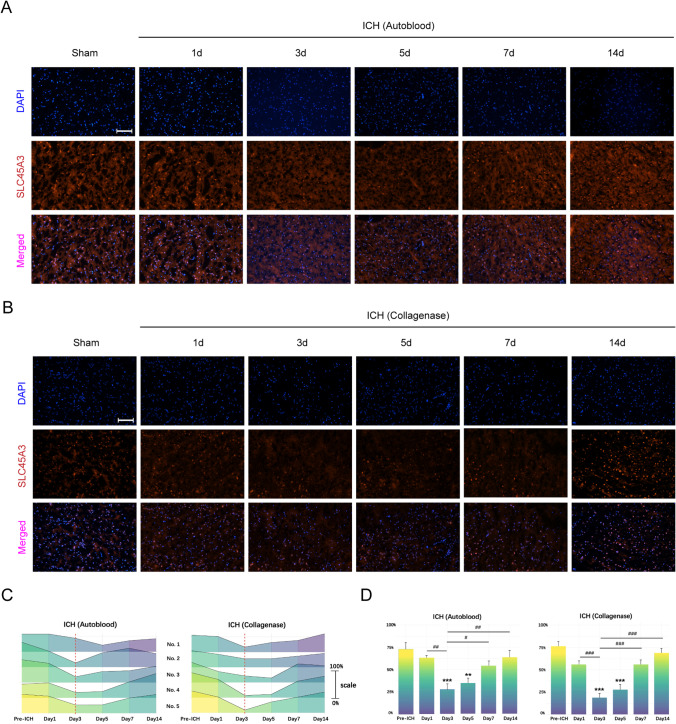
Protein expression levels of gene SLC45A3 after ICH. **A**, **B** Results of immunofluorescence staining in two models. **C** Expression levels of each mouse in two models at different time points after ICH. The height of ridges represents the ratio compared with sham group, and the vertical red dashed lines shows day 3 after ICH (*n* = 5). **D** Analysis of statistical analysis in two groups illustrated by bar charts. (*n* = 5). *P < 0.05 versus sham; **P < 0.01 versus sham; *** *P* < 0.001 versus sham; #*P* < 0.05 versus day 3; ##*P* < 0.01 versus day 3; ###*P* < 0.001 versus day 3

To investigate the changes of SLC45A3 after ICH, the expression levels were explored within brains at different time points after ICH. It is demonstrated that after ICH, the protein expression was decreased, compared to that of the sham group. The most evident decreasing trend could be observed at day 3 in both two models (Fig. 4A–D). This further suggests that SLC45A3 may play a significant role in ICH-induced brain damage.

### Overexpression of SLC45A3 Ameliorated Brain Injury After ICH

**Fig. 5:**
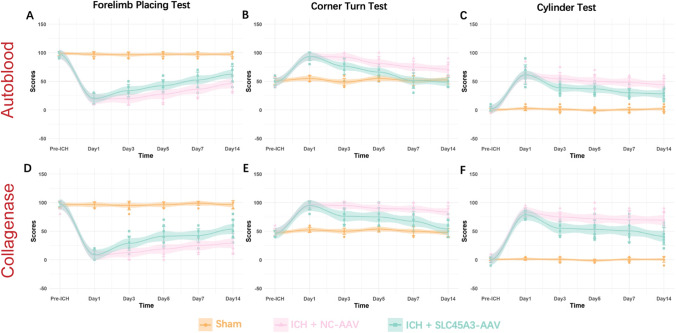
Assessments of neurobehavioral function among three groups at different time points. **A**, **D** Forelimb placing test in two models (*n* = 8). **B**, **E** Corner turn test in two models (*n* = 8). **C**, **F** Cylinder test in two models (*n* = 8). Colors represent different groups (the sham, ICH + NC-AAV and ICH + SLC45A3-AAV groups), and the shadows show the mean ± SD along with the fitted lines. *P < 0.05 versus sham; **P < 0.01 versus sham; ***P < 0.001 versus sham; #*P* < 0.05 versus ICH + NC-AAV, ##*P* < 0.01 versus ICH + NC-AAV, ###*P* < 0.001 versus ICH + NC-AAV. The colors of lines, dots, and symbols (* and #) denote different groups, as illustrated at the bottom of the figure

Western blotting was conducted to determine the effect of SLC45A3-AAV in mice. The outcome suggests that after 3 weeks, SLC45A3-AAV treatment pronouncedly increased the protein levels of SLC45A3, compared with NC-AAV group (Supplementary Fig. 3). To investigate the potential role of SLC45A3 in ICH-induced brain injury, furthermore, we assessed and compared neurofunctions among the sham, ICH + NC-AAV and ICH + SLC45A3-AAV groups. In the forelimb placing test, in comparison with the ICH + NC-AAV group at day 3, the ICH + SLC45A3-AAV group exhibited a distinct increment of the scores for the right forelimbs (Fig. 5A, D). The corner turn test showed a better balance between left and right in the ICH + SLC45A3-AAV group than the ICH + NC-AAV group at day 3 (Fig. 5B, E). As for cylinder test, a lower percentage can be observed in the ICH + SLC45A3-AAV group in contrast to the ICH + NC-AAV group at day 3 (Fig. 5C, F), indicating a better neuronal function.

### Overexpression of SLC45A3 Ameliorates the WMI After ICH

**Fig. 6:**
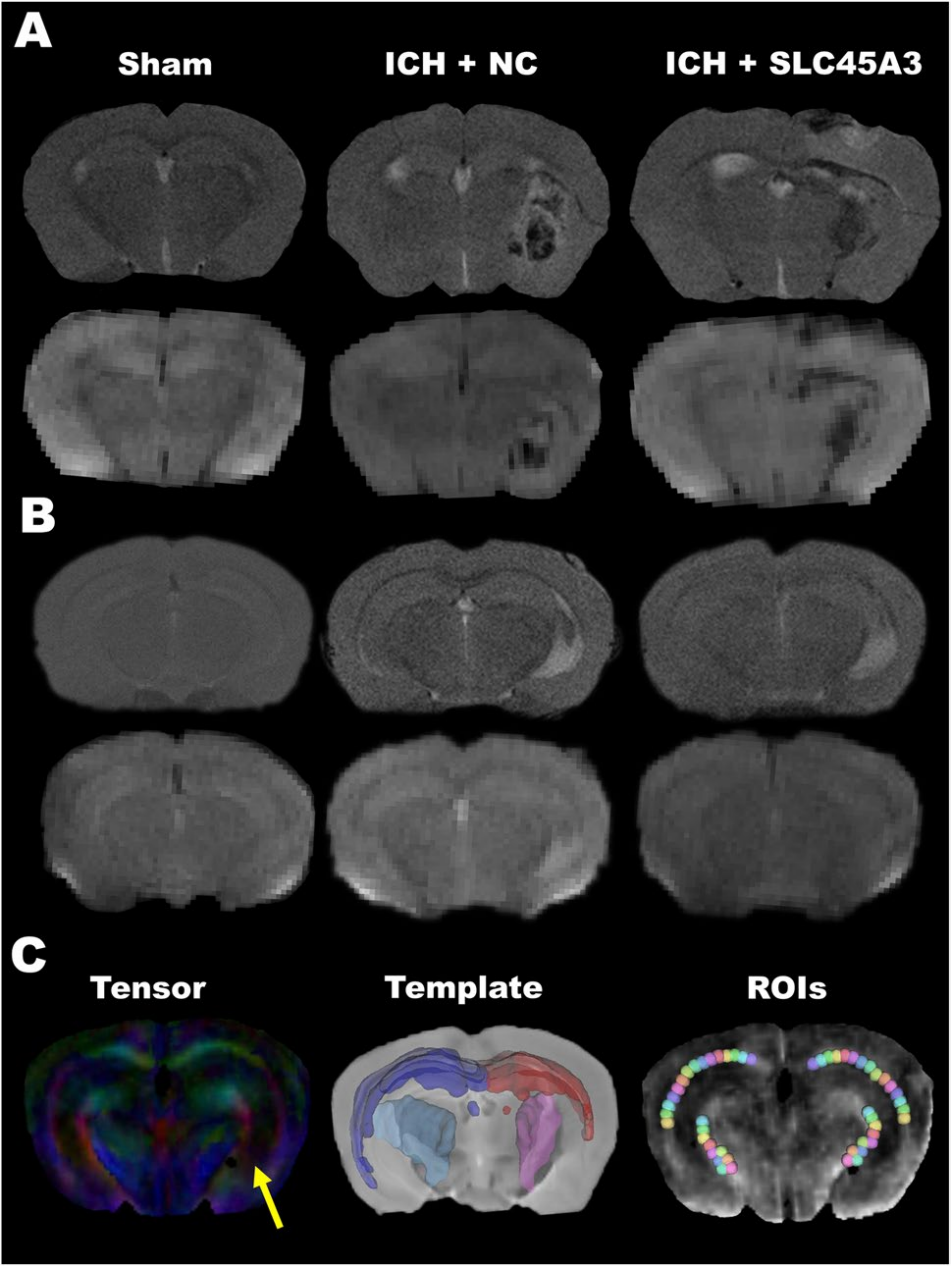
Typical MRI data of three groups in both two models. **A** T2W images (upper row) and DWI (lower row) in autologous whole blood models. **B** T2W images (upper row) and DWI (lower row) in collagenase models. **C** Colored FA map (left), registered standard template (middle), and defined ROIs (right). The yellow arrow indicates the damage in right internal capsule

According to the outcomes above, MRI was scanned 3 days after ICH. Figure 6A exhibits typical T2-weighted images (upper row) and diffusion-weighted images (lower row) of the three groups: sham, ICH + NC-AAV, and ICH + SLC45A3-AAV groups (in autologous blood models). The hematoma can be markedly spotted in the right side whereas no lesion can be recognized in sham groups. Figure 6B displays the same type of data as 6A but in collagenase models. Figure 6C illustrates the colored FA map, registered standard template, and carefully defined ROIs. In colored FA map (from ICH group), the direction of tensor in three-dimensional space was expressed by the color. By comparing the structure of right and left sides, it can be found that the structures of CC in both sides were rather undamaged, whereas that of IC in the right side was no longer intact (indicated by the yellow arrow). Both standard CC and IC of two sides were overlaid on registered template. These structures were not perfectly correctly matching the real positions, and therefore, we carefully defined ROIs, with voxel size and relative locations taken into consideration as explained in the “Methods and materials.”

**Fig. 7:**
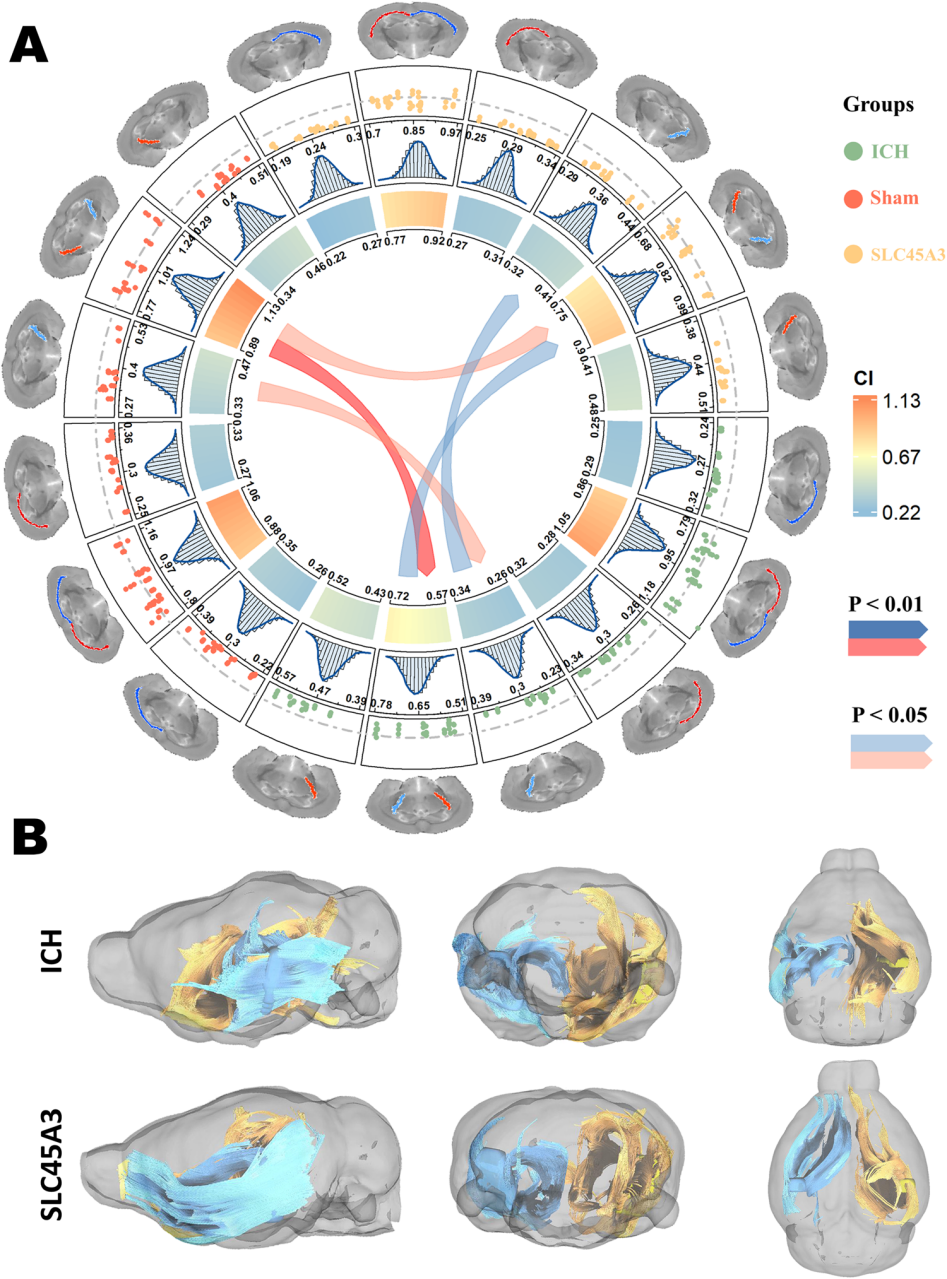
Results of statistical analysis of FA values and fiber tracking in autologous whole blood injection models. **A** In this complex circle, the outset image layer indicates the type of data. For example, starting from the 3 o’clock position clockwise, FA for right CC, FA ratio for CC, FA for left CC, FA for right IC, FA ratio for IC, and FA for left IC were showed in sequence. The second layer presents raw FA data from ROIs through a scatter plot, in which the middle gray dotted line means the value 1, and three groups (sham group, ICH for ICH + NC-AAV group, SLC45A3 for ICH + SLC45A3-AAV group) were distinguished through different colors. The third layer illustrates the mean FA values from 10,000 times resampling by bootstrap method through histogram, in which the blue curves reveal fitting outcomes of distributions, and scale marks exemplify the ranges. The fourth layer demonstrates the 95% confidence intervals of each subgroup according to their resampling outcomes, and the colors denote the amounts, and scale marks represent the exact numbers. In the center, the arrow links display statistically significant difference through two samples permutation test, in which red arrow means the value from the bottom is higher than that from the head, vice versa for blue arrow (*n* = 3). **B** Example fiber tracking results of ICH + NC-AAV group (upper row) and ICH + SLC45A3-AAV group (lower row) in three-dimensional views. Blue fibers are those from perihematomal areas whereas yellow ones are from contralateral counterpart

Basic FA values of autologous whole blood injection models and statistical analysis results of all 18 subgroups are exhibited in Fig. 7A. From the outset layer to the center, data type, FA values from ROIs, mean FA values from bootstrap resampling, 95% confidence intervals (CI), and statistically significant differences between subgroups are illustrated in sequence. Since the FA is a normalized index ranging from 0 to 1, the ones in all subgroups are under the middle gray dotted line. The FA ratios fluctuate with the line, except for those of IC, which are noticeably below it. Although no distinct difference was found among three groups in CC, significant decrease of FA values and ratios was observed in ICH + NC-AAV and ICH + SLC45A3-AAV group (henceforth abbreviated as ICH and SLC45A3 in figures, respectively), compared with the sham group. And the same decline also can be discovered in ICH group, in contrast with SLC45A3 group. Figure 7B suggests more fibers remained in SLC45A3 group, or rather, less white matter injury (WMI).

The data of same type but in collagenase injection models are illustrated in Supplementary Fig. 4. The trend is the same as in autologous blood models that FA values and ratios are higher than the other groups in IC, as well as FA values in left and right CC. The differences between the ratios of ICH + NC-AAV and ICH + SLC45A3-AAV group, however, are not significant neither in CC nor IC. And other decreases are observed in FA values of ICH + NC-AAV group in right IC and CC of both sides, compared with ICH + SLC45A3-AAV group.

**Fig. 8:**
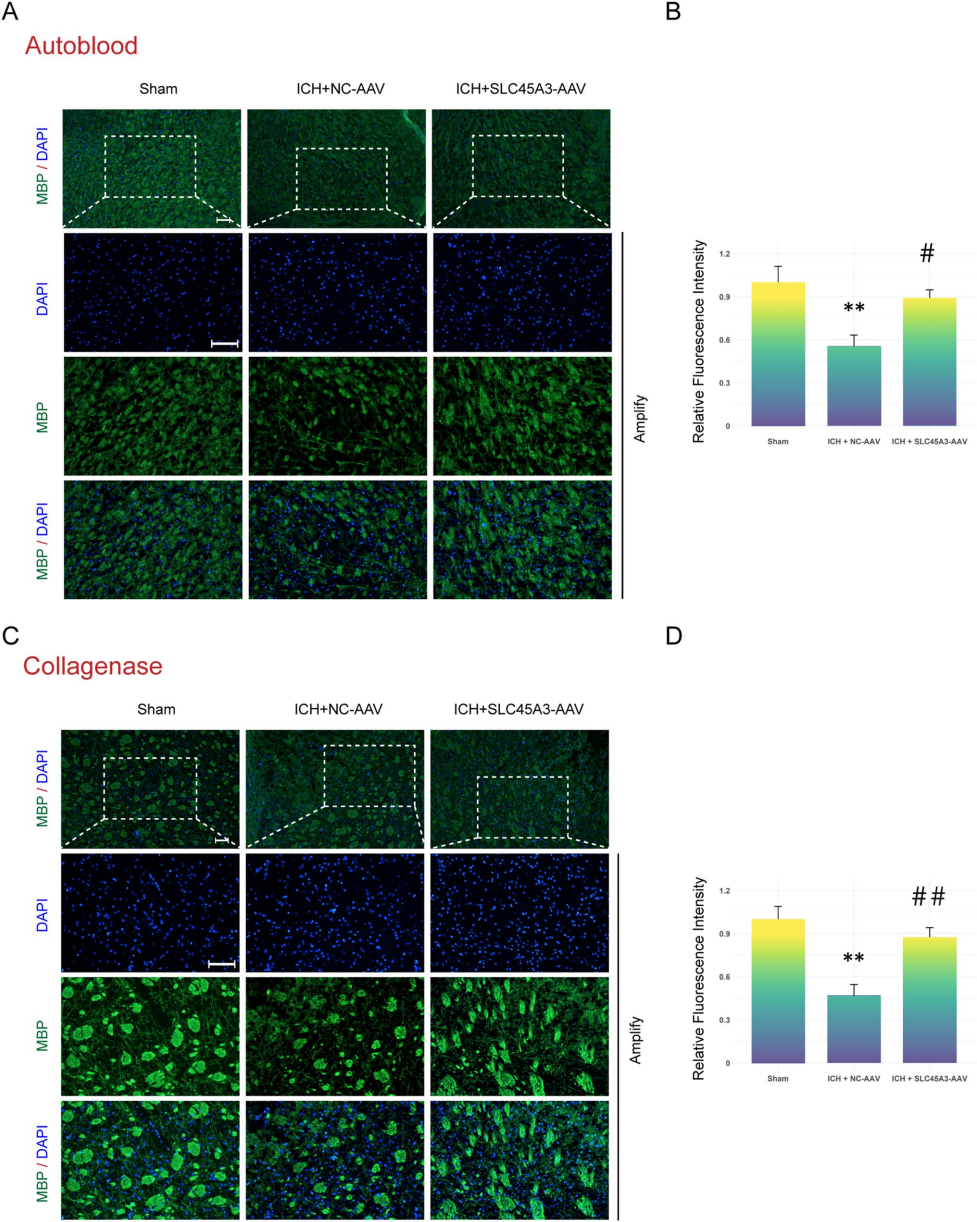
The results of immunofluorescence staining experiments. **A**, **B** Photos of immunofluorescence staining and statistical outcomes in autologous whole blood injection models. **C**, **D** Photos of immunofluorescence staining and statistical outcomes in collagenase injection models. *n* = 5. **P < 0.01 versus sham; #*P* < 0.05 versus ICH + NC-AAV, ##*P* < 0.01 versus ICH + NC-AAV

The results of immunofluorescence staining, furthermore, demonstrated that overexpression of SLC45A3 can prevent the decrease in the mean fluorescence intensity of MBP in the peri-hematoma region at day 3 after ICH (Fig. 8A–D), suggesting that the overexpression of SLC45A3 attenuated WMI induced by ICH.

## Discussion

ICH is a serious disease with high mortality and disability rates, which impairs patients’ white matter and causes neurological dysfunctions even after timely clinical interventions [37]. Increasing studies indicate that modulating WMI could pronouncedly attenuate neurological dysfunctions after ICH [12]. Some research aimed at investigating the changes of gene expression profile after ICH in rodents [38] and humans [4, 5]. In our study, by taking an intersection among interesting genes found in datasets, gene SLC45A3 was identified as the target gene. As for the other two genes (CMTM5 and COL9A2), the former is related to chemokine-like factor superfamily while the latter is associated with type IX collagen. After literature reviews, one study recently published this year reported that CMTM5 involves in sustaining axonal integrity [39], whereas no research about these two genes on white matter was found before we started our experiment; therefore, we turned our attention to SLC45A3 in our study.

The gene SLC45A3 downregulated both in the perihematomal tissues and in the peripheral blood compared with that at the third day after ICH. Through GO and KEGG analysis, it is suggested that gene SLC45A3 plays a role in processes, such as regulation of oligodendrocyte and glial cell differentiation, fatty acid biosynthetic process, gliogenesis, and positive regulation of fatty acid biosynthetic process. “The Human Protein Atlas” [40] exhibited that in human brain, SLC45A3 is mostly expressed in oligodendrocytes, next in excitatory neurons, oligodendrocyte precursor cells, astrocytes, and inhibitory neurons, which is consistent with the results of our scRNA-seq analysis. SLC45A3, or rather, solute carrier family 45 member 3, is predicted to be located in plasma membrane and biased expression in prostate. Previous research on SLC45A3 mainly focused on prostate cancer, revealing that its expression was heterogeneous and was highly induced in prostate cancers of certain subset [41–43]. In brain, Shin et al. illustrated that miR-32 and its downstream target (SLC45A3) played a vital role in myelin maintenance via regulating lipid metabolism and myelin protein expression in oligodendrocytes in cell experiments [44]. Accordingly, we hypothesized that overexpression SLC45A3 could ameliorate white matter injury after ICH.

The lipid characteristics of myelin and the presence of myelin-specific proteins are known to be critical for proper myelin structure and function. Through functional enrichment analysis, we found that SLC45A3 plays an important role in maintaining the normal structure and function of myelin, which makes a difference in the recovery of WMI. Previous research reported that SLC45A3 is a myelin-enriched putative sugar transporter that regulates myelination through its role in lipid metabolism [44]. SLC45 family proteins might be involved in the transportation of sugars (e.g., glucose and galactose). When sugar comes into cells, acetyl-CoA carboxylase will catalyze the carboxylation of acetyl-CoA to form malonyl-CoA and fuels malonyl-CoA for de novo synthesis of long-chain fatty acids. The process leads to subsequent chain elongation by fatty acid synthase and further elongation through the extension of very long–chain fatty acids proteins. These products are incorporated into neutral lipids such as cholesterol esters and phospholipids and used for steroidogenesis and energy production [45, 46]. In view of our findings and literature reviews, we further verified the role of SLC45A3 in WMI after ICH.

We established two ICH mice models induced by autologous whole blood or collagenase. These basic medical experiments showed that the expression level of SLC45A3 decreased most apparently at day 3 after ICH, and confirmed overexpression of it helped improve the rehabilitation of neurobehavioral function. These results suggest the potential capability of SLC45A3 to attenuate WMI. Since FA values have been reported to be a reliable parameter of reflecting the integrity of white matter after ICH in human [47, 48] and rodents [49], we further used DTI to detect the FA values in vivo at day 3 after ICH. In two models, they displayed the same tendency that FA values of ICH + SLC45A3-AAV group were higher than those of ICH + NC-AAV group but lower than those of sham group, coinciding with former experiments. However, in autologous blood injection groups, statistically significant difference existed in ratios of IC and FA values of left IC, whereas in collagenase injection groups, distinctions were found in not only the abovementioned subgroups but also in FA values of right IC, left CC, and right CC. We speculate the disparity was resulting from the different influence of autologous blood and collagenase to brain (that was, collagenase caused more extensive injury compared with the localized damage deriving from blood [50, 51]), which consequently devastated the white matter in CC and contralateral IC. Additionally, as is illustrated by the results of immunofluorescence staining, overexpression SLC45A3 facilitates the restoration of MBP levels (a marker reflecting white matter recovery [52]). In brief, from corresponding animal experiments in vitro and in vivo, we furtherly verified the function of SLC45A3 in ICH-induced WMI.

There are still some limitations in our study. Firstly, we only investigated the function of SLC45A3 in the difference of phenotype. More research about the exact mechanism resulting in this phenomenon should be conducted in the future. Some possible ones may be regulation of fatty acid biosynthetic process and oligodendrocyte and glial cell differentiation. Secondly, although we verified the function of SLC45A3 by multiple experiments via more accurate statistical methods, our experiments were defective: no knockdown models were included, and immunofluorescence staining cannot precisely determine the expression level; morphological and ultrastructural evidence (e.g., transmission electron microscopy provides more direct proof of WMI after ICH [53]) could be adopted. Thus, it is worthwhile to investigate the mechanism with more careful experimentation in future study.

## Conclusion

In conclusion, with the help of advanced bioinformatical algorithm, we found the gene SLC45A3 as our target gene by intersecting interesting genes, and further basic medical experiments and DTI verified overexpression of SLC45A3 may ameliorate WMI after ICH, contributing a fruitful foundation in WMI after ICH to future research.

### Supplementary Information

Below is the link to the electronic supplementary material.

Supplementary file1 (DOCX 2071 KB)

## Data Availability

All raw data used in this manuscript are available on reasonable request.

## Abbreviations

AAV: Adeno-associated virus
CC: Corpus callosum
DEGs: Differentially expressed genes
DTI: Diffusion tensor imaging
FA: Fractional anisotropy
GEO: Gene Expression Omnibus
GO: Gene Ontology
GS: Gene significance
IC: Internal capsule
ICH: Intracerebral hemorrhage
KEGG: Kyoto Encyclopedia of Genes and Genomes
MBP: Myelin basic protein
MEs: Module eigengenes
ROIs: Regions of interests
scRNA-seq: Single-cell RNA sequencing
SLC45A3: Solute Carrier Family 45 Member 3
SVM: Support vector machine
WGCNA: Weighted gene co-expression networks analysis
WMI: White matter injury
