# Supplementary material for: SLC45A3 Serves as a Potential Therapeutic Biomarker to Attenuate White Matter Injury After Intracerebral Hemorrhage
Source: Transl Stroke Res. 2023 Mar 13;15(3):556–71. doi: 10.1007/s12975-023-01145-5 (PMC11106206; doi:10.1007/s12975-023-01145-5)
Supplement: Supplementary file 1 — Supplementary file1 (DOCX 2071 KB) [file 12975_2023_1145_MOESM1_ESM.docx]

**Supplementary Figure 1.**


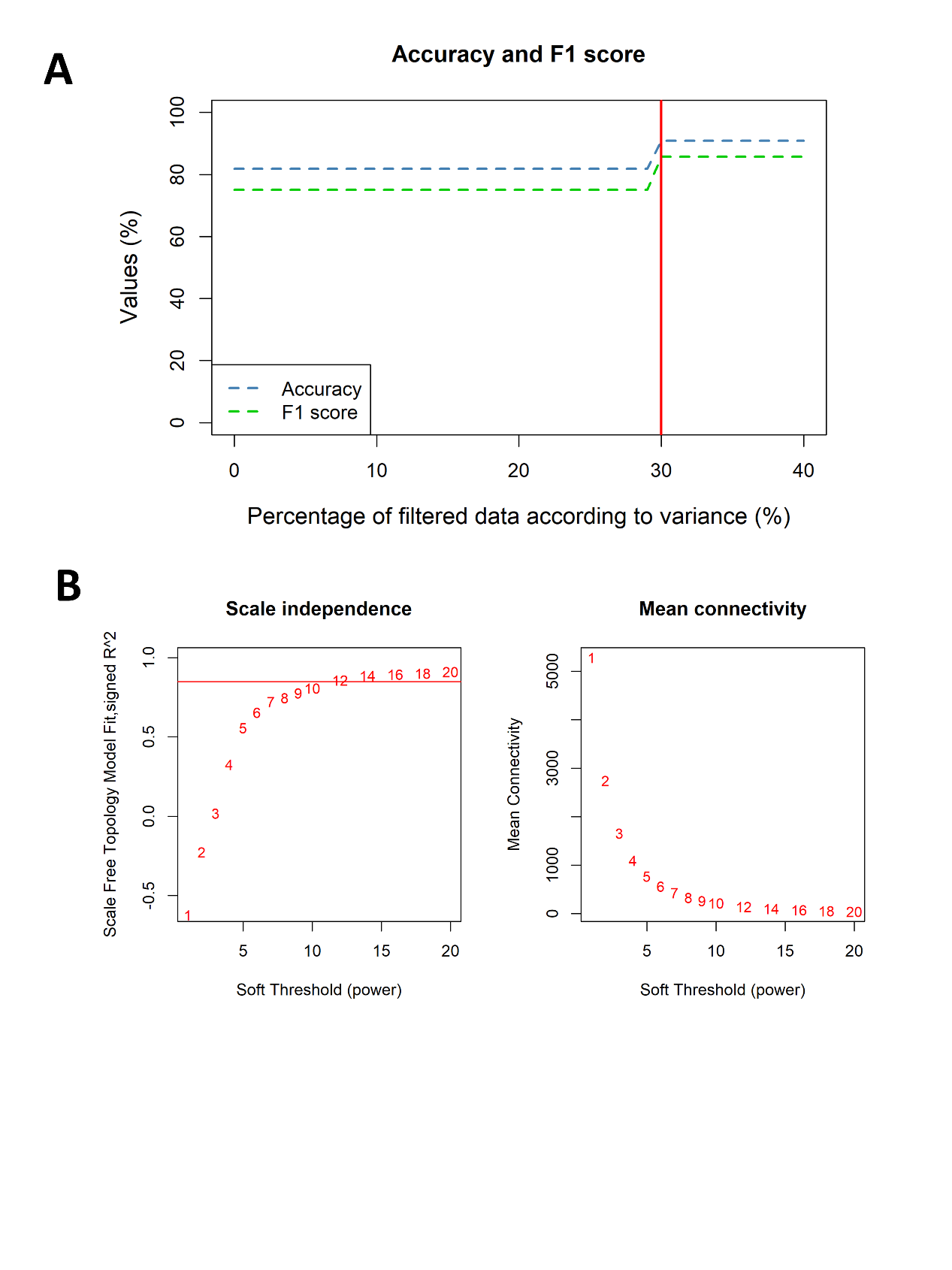


Supplementary Figure 1.

(A) Results of the performance measures of SVM. (B) The soft thresholding power β determination (left) and mean connectivity (right).

**Supplementary Figure 2.**


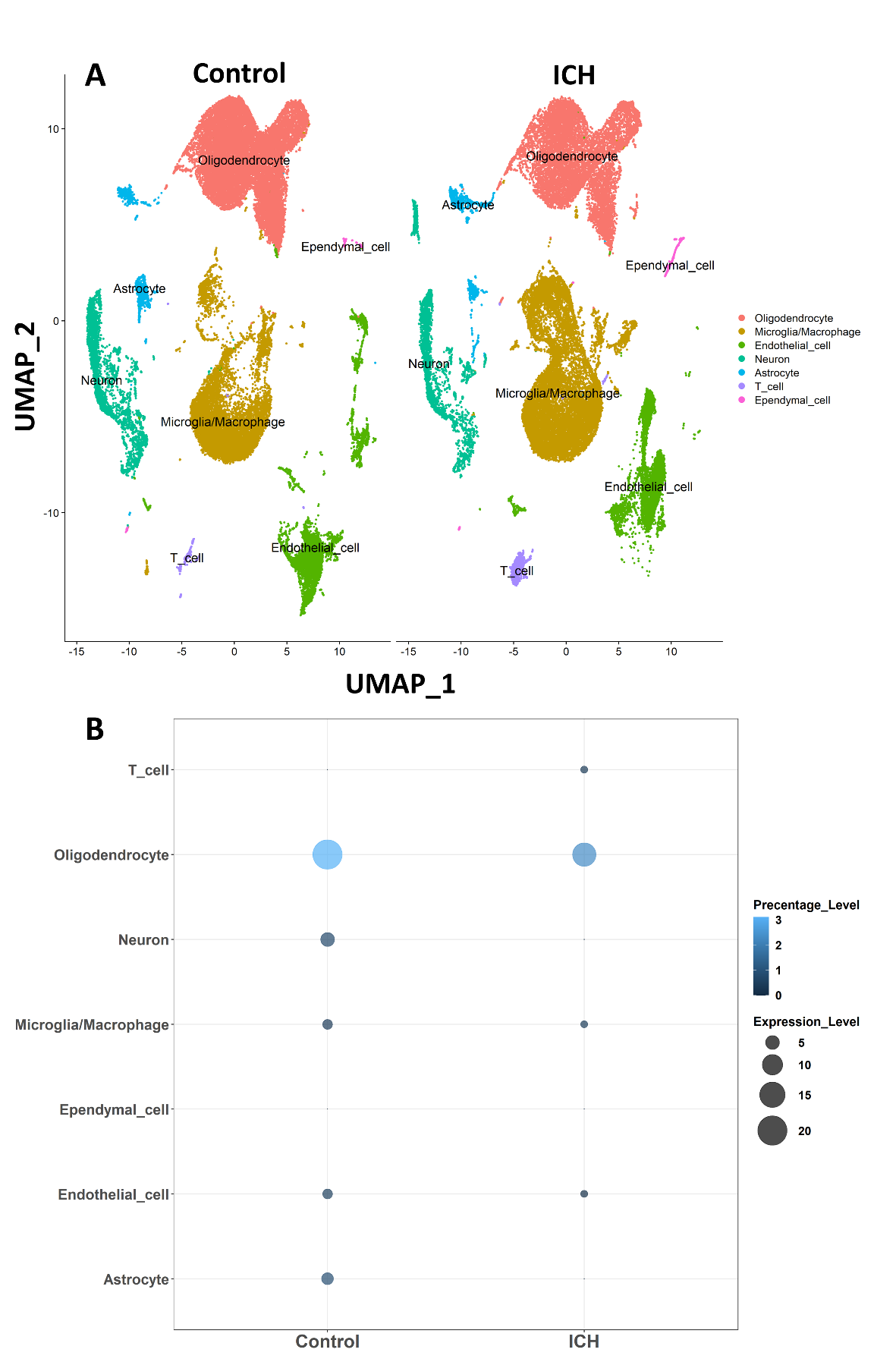


Supplementary Figure 2.

(A) Comparation of cluster analysis through UMAP. (B) Comparation of the expression of SLC45A3 in 7 cell types

**Supplementary Figure 3.**


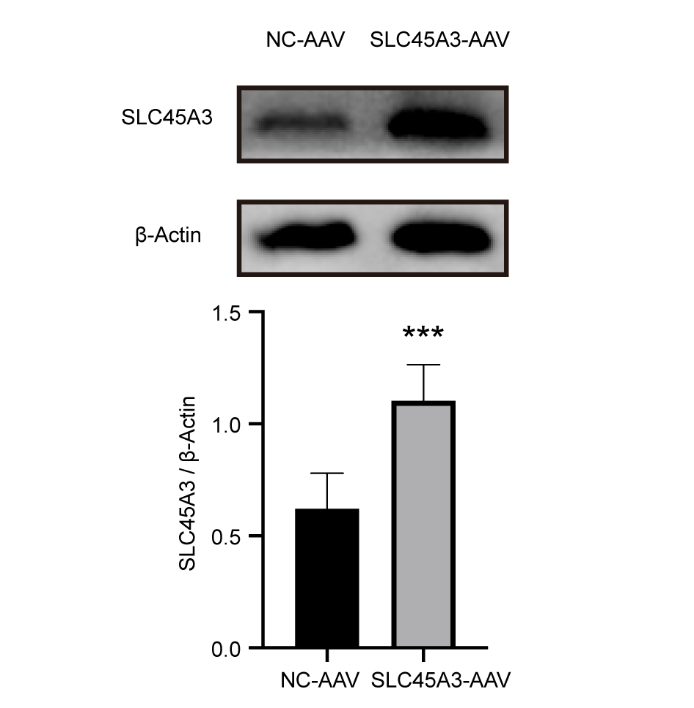


Supplementary Figure 3.

Representative western blotting images and quantitative analyses of SLC45A3 expression in brain. n = 6. *** P < 0.001 versus NC- AAV.

**Supplementary Figure 4.**


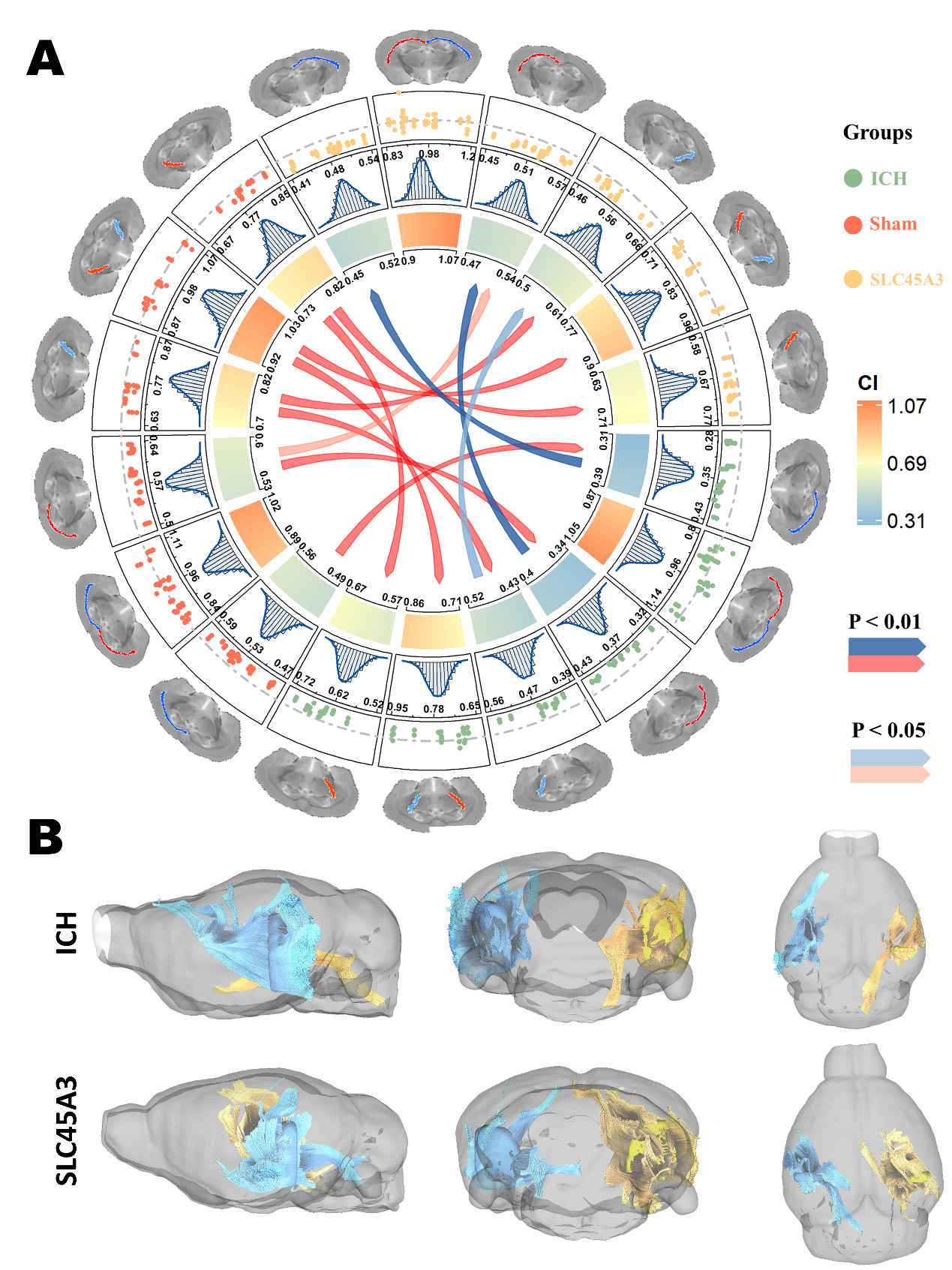


Supplementary Figure 4.

Results of statistical analysis of FA values and fiber tracking in collagenase injection models. (A) FA values of all 18 subgroups and corresponding results of statistical analysis. n = 8. (B) Example fiber tracking results of ICH + NC-AAV group (upper row) and ICH + SLC45A3-AAV group (lower row) in three views. Blue fibers are those from perihematomal areas whereas yellow ones are from contralateral counterpart.
